# Effects of multiple-dose intranasal oxytocin administration on social responsiveness in children with autism: a randomized, placebo-controlled trial

**DOI:** 10.1186/s13229-023-00546-5

**Published:** 2023-04-20

**Authors:** Nicky Daniels, Matthijs Moerkerke, Jean Steyaert, Annelies Bamps, Edward Debbaut, Jellina Prinsen, Tiffany Tang, Stephanie Van der Donck, Bart Boets, Kaat Alaerts

**Affiliations:** 1 Department of Rehabilitation Sciences, KU Leuven, Tervuursevest 101, Box 1501, 3001 Leuven, Belgium; 2 Leuven Autism Research (LAuRes), KU Leuven, Leuven, Belgium; 3 Department of Neurosciences, KU Leuven, Leuven, Belgium; 4 Department of Child Psychiatry, UPC KU Leuven, Leuven, Belgium

**Keywords:** Autism spectrum disorder (ASD), Oxytocin, Social responsiveness, Randomized controlled trial

## Abstract

**Background:**

Intranasal administration of oxytocin is increasingly explored as a new approach to facilitate social development and reduce disability associated with a diagnosis of autism spectrum disorder (ASD). The efficacy of multiple-dose oxytocin administration in children with ASD is, however, not well established.

**Methods:**

A double-blind, randomized, placebo-controlled trial with parallel design explored the effects of a 4-week intranasal oxytocin administration (12 IU, twice daily) on parent-rated social responsiveness (Social Responsiveness Scale: SRS-2) in pre-pubertal school-aged children (aged 8–12 years, 61 boys, 16 girls). Secondary outcomes included a questionnaire-based assessment of repetitive behaviors, anxiety, and attachment. Effects of oxytocin were assessed immediately after the administration period and at a follow-up, 4 weeks after the last administration. The double-blind phase was followed by a 4-week single-blind phase during which all participants received intranasal oxytocin.

**Results:**

In the double-blind phase, both the oxytocin and placebo group displayed significant pre-to-post-improvements in social responsiveness and secondary questionnaires, but improvements were not specific to the intranasal oxytocin. Notably, in the single-blind phase, participants who were first allocated to intranasal placebo and later changed to intranasal oxytocin displayed a significant improvement in social responsiveness, over and above the placebo-induced improvements noted in the first phase. Participants receiving oxytocin in the first phase also showed a significant further improvement upon receiving a second course of oxytocin, but only at the 4-week follow-up. Further, exploratory moderator analyses indicated that children who received psychosocial trainings (3 or more sessions per month) along with oxytocin administration displayed a more pronounced improvement in social responsiveness.

**Limitations:**

Future studies using larger cohorts and more explicitly controlled concurrent psychosocial trainings are warranted to further explore the preliminary moderator effects, also including understudied populations within the autism spectrum, such as children with co-occurring intellectual disabilities.

**Conclusions:**

Four weeks of oxytocin administration did not induce treatment-specific improvements in social responsiveness in school-aged children with ASD. Future studies are warranted to further explore the clinical efficacy of oxytocin administration paired with targeted psychosocial trainings that stimulate socio-communicative behaviors.

*Trial registration* The trial was registered with the European Clinical Trial Registry (EudraCT 2018-000769-35) on June 7th, 2018 (https://www.clinicaltrialsregister.eu/ctr-search/trial/2018-000769-35/BE).

**Supplementary Information:**

The online version contains supplementary material available at 10.1186/s13229-023-00546-5.

## Background

Autism spectrum disorder (ASD) is a neurodevelopmental condition characterized by impairments in social communication and interaction, combined with restricted and repetitive behaviors and interests [1]. While a variety of behavioral, pharmacological, and environmental interventions have been put forward to enhance the quality of life and reduce the disabilities associated with an autism diagnosis [2, 3], for many individuals, significant enduring disability remains. In this context, several clinical trials have examined intranasal administration of oxytocin (OT) as a new approach to facilitate social development and reduce disability [4]. OT is an endogenous neuropeptide that is mainly produced in paraventricular nuclei of the hypothalamus. In the brain, OT acts as an important neuromodulator for a broad range of affiliative and prosocial behaviors, including interpersonal bonding, social attunement and attachment [5–7], presumably mediated through its postulated top-down enhancing effect on ‘social salience’ and bottom-up effect on regulating (social) stress and anxiety [8, 9].

Following a myriad of single-dose proof-of-principle studies [4], an initial multiple-dose pilot study assessed the safety and efficacy of 6 weeks of chronic intranasal OT administration in 19 autistic adults (10 receiving 24 IU 2x/day of OT, 9 receiving placebo) and showed improved emotion recognition and quality of life, and tentative improvements in repetitive behaviors after OT administration [10]. Later, significant improvements on the Clinical Global Impression-Improvement scale after 12 weeks of OT administration in adult men with ASD were shown, albeit only in the subgroup of participants receiving the high-dose nasal spray administration (32 IU 1x/day; *n* = 13), and not in the low-dose (16 IU 1x/day; *n* = 15) or placebo groups (*n* = 16) [11]. In an exploratory crossover study [12], the effects of 6 weeks of daily intranasal OT administration were studied in 18 adult men with ASD (9 receiving 24 IU 2x/day of OT, 9 receiving placebo), and significant improvements in social reciprocity and social functioning (social-judgment task) were identified. A confirmatory trial with an identical protocol as in [12] in 106 adult men with ASD (53 receiving 24 IU 2x/day of OT, 53 receiving placebo) similarly identified significant improvements in repetitive behaviors, but the effects on social reciprocity and social functioning could not be replicated [13]. These observations were further extended in an exploratory sample of 40 young adult men with ASD (22 receiving 24 IU 1x/day of OT, 18 receiving placebo), demonstrating long-term improvements in repetitive behaviors and feelings of attachment after a 4-week course of OT administration, with improvements outlasting the period of administration till 1 year post-treatment [14].

Given that autism is an early-onset neurodevelopmental condition, it is important to extend these insights to pediatric populations, allowing evaluations of the efficacy of OT administration within an early developmental window and whether it can be facilitatory for enriching social behaviors and experiences from an early age onwards. To date, a handful of trials explored the effects of multiple-dose OT administration in children with ASD. Two initial trials reported a consistent pattern of results, indicating improvements in the social domain (parent-reported social responsiveness) after 5 weeks of OT administration (12 IU 2x/day) in 3- to 6-year-old children with ASD (*n* = 31, crossover [15]) and after 4 weeks of OT administration in 6- to 12-year-old children with ASD (14 receiving 24 IU 2x/day of OT, 18 receiving placebo [16]). No significant improvements on outcomes of social function or repetitive behaviors were demonstrated, however, after an 8-week OT administration period in adolescent boys with ASD (26 receiving 18 or 24 IU 2x/day of OT, 24 receiving placebo, 12–18 years [17]), or in a preliminary 12-week administration trial encompassing a broad age range of 5- to 17-year-old children (8 receiving 12 IU 1x/day of OT, 10 receiving placebo) with Phelan–McDermid syndrome (characterized by ASD-like phenotypes [18]). Also, in a recent confirmatory trial including 3- to 17-year-old children with ASD (139 OT / 138 placebo) and an age-adjusted dosing scheme ranging from a daily dose of 8–80 IU, no improvements on outcomes of social functioning were evident after 24 weeks of OT administration [19].

Several factors have been put forward to understand these inconsistent results, ranging from heterogeneity in trial design (e.g., parallel versus crossover design, adopted outcomes, dosing scheme) to variation in participant characteristics. For instance, the above-mentioned well-powered confirmatory trial covered a broad age range (3–17 years) [19], encompassing a critical period of pubertal development, which could have rendered heterogeneity due to differential physiologic effects of OT during different developmental stages [20].

Here, results are presented from a single-center, randomized, double-blind, placebo-controlled clinical trial (RCT with parallel design), testing efficacy on outcomes of social functioning and safety of multiple-dose OT administration (4 weeks of twice daily intranasal administration of 12 IU) in a representative sample of 8- to 12-year-old children with ASD (40 OT/40 placebo). Accordingly, by examining OT administration effects in a relatively strict age range of pre-pubertal, school-aged children, the current trial particularly aimed to minimize the potential influence of sample heterogeneity on treatment outcome. Further, following prior observations of long-lasting retention effects of OT administration in adults with ASD [14], the current trial similarly included a follow-up session 4 weeks after cessation of the daily OT administrations, testing the possibility of crucial retention effects in the current pediatric sample. After completing the follow-up session, participants were further enrolled in a second ‘single-blind’ phase (phase II), allowing the placebo-first group to also receive the active OT nasal spray and the oxytocin-first group, to receive a second 4-week course of OT administration [14].

Finally, a series of exploratory analyses were conducted to examine whether individual variation in treatment response may relate to particular moderator variables. First, considering prior notions that expectancies about allocated treatment can impact treatment outcome [17], parental belief about allocated nasal spray (oxytocin versus placebo) was assessed and adopted as a potential moderator variable. Second, considering prior evidence of differential effects of oxytocin administration in men and women [21], we investigated treatment effect moderation depending on the biological sex of the participant. Further, the impact of other person-dependent variables, such as co-occurring conditions, medication use and ongoing engagement in psychosocial therapies during the OT administration period, was tested as possible moderator variables. The latter factor was included in light of recent notions that the context of nasal spray administration may impact the efficacy of oxytocin, especially if it is a socially stimulating context [22, 23].

## Methods

### General study design

**Fig. 1:**
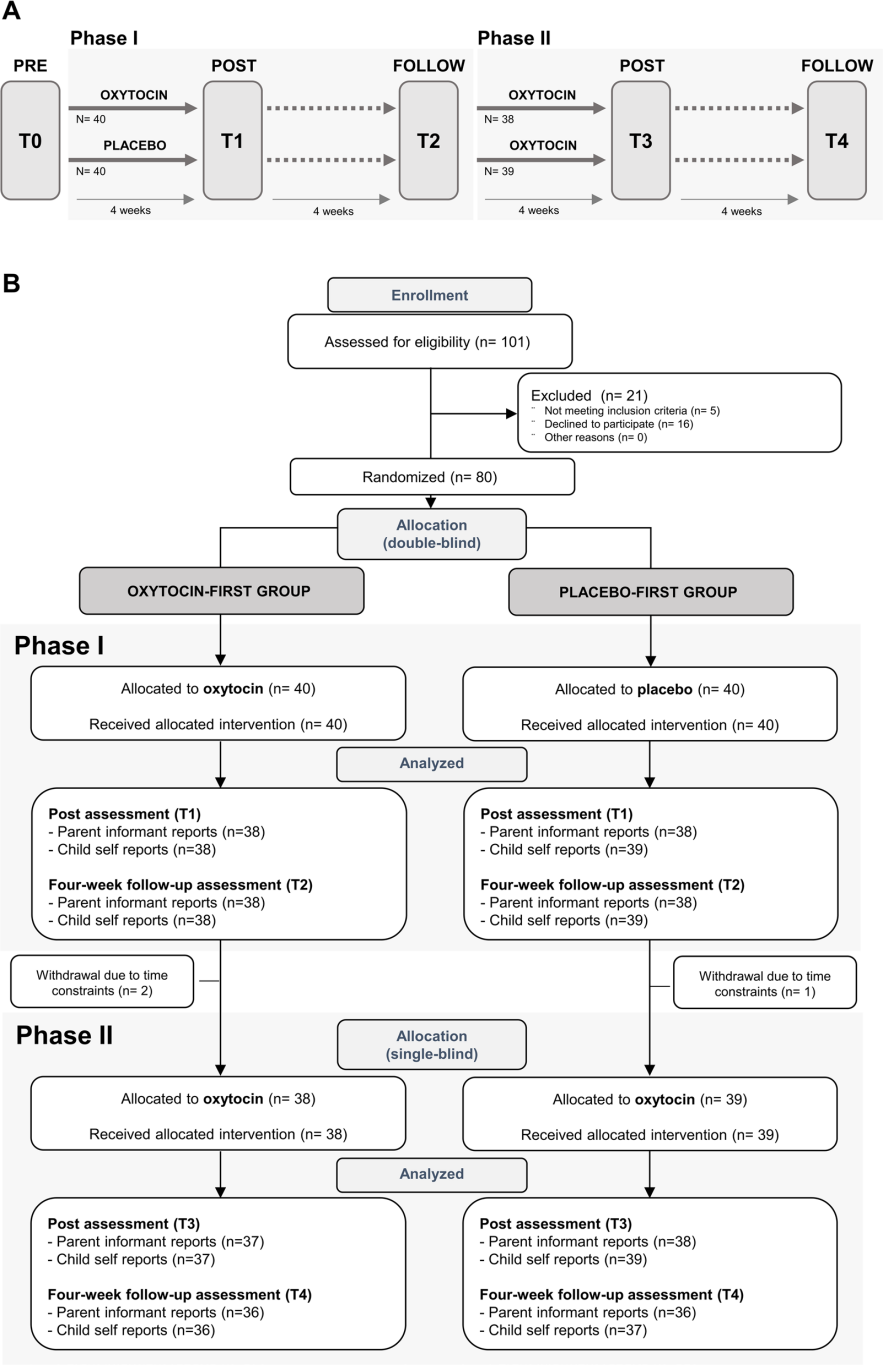
Trial design (panel **A**) and CONSORT flow diagram of participants in the trial (panel **B**). Participants first underwent a double-blind phase (phase I) during which they were allocated to administer either oxytocin or placebo (4 weeks of twice daily intranasal administration). In phase I, nasal spray administration effects were assessed immediately after the last administration of the 4-week administration period (post, T1) and at a follow-up session, four weeks after cessation of the daily administrations (follow-up, T2). Phase I was immediately followed by a single-blind phase (phase II, during which all participants received four weeks of intranasal oxytocin. Also in phase II, nasal spray administration effects were assessed immediately after the four-week administration period (post, T3) and at a follow-up session, four weeks after cessation of the daily administrations (follow-up, T4) (panel **A**). The CONSORT flow diagram (panel **B**) visualizes the number of participants throughout the trial, indicating completed assessments at each session, separately for parent informant- and child self-reports

The RCT with a parallel design assessing the effect of multiple-dose OT administration in children with ASD was performed at the Leuven University Hospital (Belgium). The double-blind phase (phase I) was followed by a 4-week single-blind phase (phase II) during which all participants received intranasal OT. In both phases, OT administration effects were assessed immediately after the 4-week administration period (i.e., post-measurement T1 and T3) and at a follow-up session 4 weeks after cessation of the daily administrations (i.e., follow-up measurement T2 and T4). Please see Fig. 1A, for a visualization of the trial design and Fig. 1B for the CONSORT Flow diagram visualizing the number of participants randomized and analyzed. Please also see Additional file 1 outlining in more detail the trial design and the impact of COVID-19-related health restrictions on the recruitment and flow of participants in the trial.

Written informed consent from the parents and assent from the child were obtained prior to the study. Consent forms and study design were approved by the Ethics Committee for Biomedical Research at the University of Leuven, KU Leuven (S61358) in accordance with The Code of Ethics of the World Medical Association (Declaration of Helsinki). The trial was registered at the European Clinical Trial Registry (EudraCT 2018-000769-35) and the Belgian Federal Agency for Medicines and Health products. As indicated in the EudraCT registration, behavioral data collections were part of a broader assessment, including (neuro)physiological and biological assessments (reports in preparation). The trial was monitored by the Clinical Trial Center at the University Hospital of Leuven, and all trial staff had Good Clinical Practice certification and was trained in the study protocol.

### Participants

Children with a formal diagnosis of ASD were recruited through the Autism Expertise Centre at the Leuven University Hospital between July 2019 and January 2021. The diagnosis was established by a multidisciplinary neuropediatric team based on the strict criteria of the DSM-5 (Diagnostic and Statistical Manual of Mental Disorders) [1]. Prior to randomization, the Autism Diagnostic Observation Schedule (ADOS-2) [24] and estimates of intelligence (four subtests of the Wechsler Intelligence Scale for Children, Fifth Edition, Dutch version) [25] were acquired (Table 1). The performance intelligence quotient (IQ) was derived from the subtests Block Design and Figure Puzzles. The verbal IQ was derived from the subtests Similarities and Vocabulary.

**Table 1:** Demographic characteristics of the trial participants at baseline (T0), separately for the oxytocin and placebo groups

	Oxytocin				Placebo				*t-*value	*p* value
	*n*	mean ± SD			*n*	mean ± SD				
Age	38	10.48 ± 1.32			39	10.39 ± 1.23			0.28	0.779
Sex	30 M/8 F				31 M/8 F					
Handedness	35 R/3 L				33 R/6 L					
WISC-V										
Verbal IQ	37	105.84 ± 14.41			38	109.42 ± 15.77			-1.03	0.308
Performance IQ	38	104.05 ± 15.36			38	101.66 ± 12.75			0.74	0.462
ADOS-2										
Total	33	9.48 ± 3.78			32	9.16 ± 4.15			0.33	0.740
Social Affect	31	7.13 ± 3.55			32	7.47 ± 3.72			-0.37	0.712
Restricted and repetitive behavior	31	2.10 ± 1.19			31	1.71 ± 1.30			1.22	0.226
SRS-2 total t-score	38	87.10 ± 13.30			39	87.10 ± 12.25			0.32	0.752
Primary outcome										
SRS-2 total raw score	38	89.26 ± 21.66			39	87.87 ± 20.03			0.29	0.771
Secondary outcomes—parent report										
RBS-R	38	27.29 ± 15.24			39	26.64 ± 16.43			0.18	0.858
SCARED parent	38	39.74 ± 21.74			39	45.15 ± 18.31			-1.18	0.240
Secondary outcomes—self-report										
SCARED child	38	38.29 ± 20.99			39	39.05 ± 20.21			-0.16	0.872
ASCQ Anxious	38	13.45 ± 5.19			39	12.85 ± 4.15			0.56	0.575
ASCQ Avoidant	38	13.79 ± 4.00			39	14.08 ± 3.86			-0.32	0.749
ASCQ Secure	38	19.97 ± 3.50			39	19.23 ± 2.78			1.03	0.305
Attachment Mother Anxiety	38	4.74 ± 2.89			39	4.82 ± 2.78			-0.13	0.897
Attachment Mother Avoidance	38	9.05 ± 4.76			39	7.97 ± 4.03			1.07	0.286
Attachment Mother Secure	38	16.76 ± 4.24			39	17.87 ± 3.13			-1.31	0.195

									Pearson Chi-square	*p* value
Co-occurring conditions	15				15				0.01	0.927
Psychoactive Medication	22				23				0.01	0.923
Psychosocial Training (*> 2 sessions/month*)	10				8				0.36	0.547

Data are shown as mean ± standard deviation. Values printed in bold are significant differences with *p*-values smaller than 0.05

*M* male, *F* female, *R* right, *L* left, *WISC-V* Wechsler intelligence scale for children, *ADOS* autism diagnostic observation schedule, *SRS-2* social responsiveness scale, *RBS-R* repetitive behavior scale-revised, *SCARED* screen for child anxiety related disorders, *ASCQ* attachment style classification questionnaire.

*Inclusion/exclusion criteria* Principal inclusion criteria comprised a clinical diagnosis of ASD, age (8–12 years old), intelligence quotient (IQ) above 70, native Dutch speaker, a stable background treatment for at least 4 weeks prior to the screening and no anticipated changes during the trial. Only premenstrual girls were included. Principal criteria for exclusion comprised any neurological (e.g., stroke, epilepsy, concussion) or significant physical disorder (liver, renal, cardiac pathology) or prior use of OT nasal spray (see Additional file 1: Table S1).

*Sample size* A total of 80 participants (40 in each treatment arm) participated in the trial, allowing to detect a medium effect size (*d* = 0.60) with *α* = 0.05 and 80% power, corresponding to effect sizes previously reported in a 4-week oxytocin trial with school-aged children [16].

*Medication use, co-occurring conditions and participation in ongoing therapies/trainings* The presence of co-occurring psychiatric conditions (with the explicit mentioning of examples in the screening interview including e.g., attention deficit hyperactivity disorder, depression, dyscalculia, dyslexia) and concurrent psychoactive medication use (defined as use within 4 weeks before study enrollment) were screened through parent-report (see Additional file 1: Table S2 for detailed information). Parents were also asked to report participation in ongoing therapies/trainings and whether these were aimed at psychosocial stimulation. Upon free report, parents indicated participation of their child in the following psychosocial trainings/therapies: Theory of Mind training, emotion recognition training, social skills training, cognitive behavioral therapy, psychotherapy, self-esteem training, mood regulation, music therapy, hippotherapy and an autism coach. To perform moderator analyses assessing the possible impact of receiving concomitant psychosocial training, the group of children was subdivided in those receiving a higher intensity of psychosocial training (3 or more sessions per month) versus those receiving no or low intensity psychosocial training (less than 3 training sessions per month) (see Table 1). Note that adopting a more lenient threshold for defining the subgroups (i.e., 1 or more session(s) per month) yielded a qualitatively similar pattern of moderator analysis results (data not shown).

### Intervention

*Study medication* Participants were randomized to receive OT (Syntocinon®, Sigma-tau) or placebo nasal sprays, administered in identical blinded amber 10-ml glass bottles with metered pump. The placebo spray consisted of all the ingredients used in the active solution except the OT compound. Nasal spray preparation, packaging, blinding and randomization (permuted-block randomization, RITA software [26]) were performed by the pharmacy of Heidelberg University Hospital (Germany). Participants were randomly assigned in a 1:1 ratio, and balanced according to sex (male/female), IQ and age. During the initial double-blind phase (phase I), all research staff conducting the trial, participants and their parents were blinded to nasal spray allocation. During the subsequent single-blind phase (phase II), trial staff were aware that all participants received intranasal OT, but participants and parents were still fully blinded regarding nasal spray allocation. Particularly, children and their parents participating in the trial were informed that during at least one of the two treatment phases, they would administer the active OT nasal spray. Only after the last visit of the last participant, trial staff were unblinded regarding treatment allocation in phase I.

*Dosing* Children (assisted by their parents) were asked to self-administer a daily dose of 2 × 12 IU nasal spray or placebo equivalent (3 puffs of 2 IU in each nostril), 12 IU in the morning and 12 IU in the afternoon (similar to the conservative dosing scheme adopted in young children with ASD [15]). The nasal spray was administered during 28 consecutive days during the initial double-blind phase (phase I) and for another 28 days during the single-blind phase (phase II). The duration of 4 weeks was similar to prior trials in children [16] and adults [14] with ASD. Participants received clear instructions about the use of the nasal sprays through a demonstration together with the trial staff [27].

*Compliance monitoring* Compliance was assured using a daily medication diary that recorded date and time of administration (phase I percentage compliance; OT: 96.75 ± 5.26%; placebo: 96.11 ± 5.29%;* t*(74) = 0.52, *p* = 0.603; phase II percentage compliance; OT-first: 94.55 ± 11.69%; placebo-first: 92.98 ± 13.92%;* t*(74) = 0.53, *p* = 0.597). The total amount of administered fluid was also monitored (phase I: OT: 14.86 ± 2.37 ml; Placebo: 13.79 ± 2.35 ml; *t*(75) = 2.00, *p* = 0.050; phase II: OT-first: 13.72 ± 3.47 ml; placebo-first: 12.83 ± 3.52 ml; *t*(74) = 1.10, *p* = 0.275).

*Side effects* During the nasal spray administration period, participants were screened for potential adverse events (weekly parent report) or changes in affect and arousal (daily diary by child and parent). Overall, reports of side effects were minimal and not treatment-specific (see Additional file 1: Tables S3 and S4).

*Parent-reported beliefs about allocated nasal spray* At the end of each trial phase (I and II), parents reported beliefs about nasal spray allocation (see Results). In the double-blind phase (phase I), the proportion of parents that believed their child had received the OT nasal spray was similar in both treatment arms: 39.5% in the OT group and 35.9% in the placebo group (*p* = 0.75). In the OT group, 18.4% of parents indicated to ‘have no explicit belief’ about nasal spray allocation versus 10.3% in the placebo group. In the single blind phase (phase II), during which all participants received the actual OT nasal spray, the proportions of parents that believed their child had received the OT nasal spray were similar as well (*p* = 0.30): 57.9% in the oxytocin-first group, 46.2% in the placebo-first group. Furthermore, the proportions of parents that believed their child had received the OT nasal spray did not differ between treatment phases (oxytocin-first: *p* = 0.11; placebo-first: *p* = 0.36).

### Outcome measures

The primary outcome measure was change from baseline in parent-rated social responsiveness on the Social Responsiveness Scale-Children, second edition (SRS-2 total raw scores) [28, 29], which comprises five subscales examining social cognition, social communication, social awareness, social motivation, and rigidity/repetitiveness, using a four-point Likert-scale (65 items). Lower scores indicate higher social responsiveness.

Secondary outcome measures included changes from baseline in parent-rated repetitive behaviors (Repetitive Behavior Scale-Revised; RBS-R) [30], self- and parent-rated presence of anxiety symptoms (Screen for Child Anxiety Related Emotional Disorders; SCARED-NL) [31], and changes from baseline in constructs of self-rated attachment toward their mother (Attachment Questionnaire child-report) [32] and peers (Attachment Style Classification Questionnaire child-report) [33] (see Table 2 and Additional file 1: Table S5).

**Table 2:** Effects of oxytocin nasal spray administration on primary and secondary outcome measures of the double-blind phase I

Outcome measure	Within-group												Between-group		
	Oxytocin group						Placebo group								
	*n*	mean ± SD			*t-*value	*p-*value	*n*	mean ± SD			*t-*value	*p-*value	Cohen's *d*	*t-*value	*p-*value
**T1 Post-assessment**															
Primary outcome															
SRS-2 total raw score	38	− 4.08 ± 10.05			− 2.50	**0.017**	38	− 4.55 ± 10.23			-2.74	**0.009**	0.05	0.20	0.839
Secondary outcomes—parent report															
RBS-R	38	− 6.53 ± 11.26			− 3.57	**0.001**	38	− 6.76 ± 9.92			− 4.20	**0.000**	0.02	0.10	0.923
SCARED Parent	38	− 0.47 ± 11.61			− 0.25	0.803	38	− 4.95 ± 11.93			− 2.56	**0.015**	0.38	1.66	0.102
Secondary outcomes—self-report															
SCARED child	38	− 4.74 ± 11.47			− 2.55	**0.015**	39	− 3.38 ± 11.32			− 1.87	0.070	− 0.12	-0.52	0.604
ASCQ secure	38	− 0.89 ± 2.42			− 2.27	**0.029**	39	− 0.92 ± 2.93			− 1.97	0.057	0.01	0.05	0.963
ASCQ anxious	38	− 0.92 ± 3.24			− 1.75	0.088	39	− 1.00 ± 2.50			− 2.50	**0.017**	0.03	0.12	0.905
ASCQ avoidant	38	− 0.29 ± 3.73			− 0.48	0.636	39	− 1.08 ± 3.30			− 2.04	**0.049**	0.22	0.98	0.330
Attachment mother anxiety	38	0.76 ± 2.95			1.59	0.120	39	− 0.33 ± 2.57			− 0.81	0.423	0.40	1.74	0.086
Attachment mother avoidance	38	− 0.21 ± 3.18			− 0.41	0.686	39	− 0.82 ± 3.94			− 1.30	0.201	0.17	0.75	0.458
Attachment mother secure	38	0.24 ± 4.18			0.35	0.729	39	− 0.18 ± 2.55			− 0.44	0.663	0.12	0.53	0.598
**T2 follow-up assessment**															
Primary Outcome															
SRS-2 total raw score	38	− 6.76 ± 11.19			− 3.73	**0.001**	39	− 5.38 ± 13.42			− 2.51	**0.017**	− 0.11	-0.49	0.626
Secondary outcomes—parent report															
RBS-R	38	− 4.55 ± 10.76			− 2.61	**0.013**	39	− 4.41 ± 8.28			− 3.33	**0.002**	− 0.01	− 0.07	0.948
SCARED parent	38	− 2.92 ± 11.53			− 1.56	0.127	39	− 5.38 ± 9.52			− 3.53	**0.001**	0.23	1.02	0.309
Secondary outcomes—self-report															
SCARED child	38	− 5.97 ± 9.84			− 3.74	**0.001**	39	− 6.36 ± 13.38			− 2.97	**0.005**	0.03	0.14	0.886
ASCQ anxious	38	− 1.00 ± 3.92			− 1.57	0.125	39	− 2.38 ± 3.75			− 3.98	**0.000**	0.36	1.58	0.117
ASCQ avoidant	38	− 0.84 ± 4.04			− 1.28	0.207	39	− 1.46 ± 3.09			− 2.95	**0.005**	0.17	0.76	0.452
ASCQ secure	38	− 1.37 ± 3.34			− 2.53	**0.016**	39	− 0.82 ± 3.78			− 1.35	0.184	− 0.15	− 0.67	0.503
Attachment mother anxiety	38	0.84 ± 3.11			1.67	0.103	39	0.08 ± 3.07			0.16	0.877	0.25	1.09	0.281
Attachment mother avoidance	38	− 0.61 ± 3.89			− 0.96	0.343	39	− 0.67 ± 3.50			− 1.19	0.241	0.02	0.07	0.942
Attachment mother secure	38	0.66 ± 3.93			1.03	0.308	39	0.13 ± 2.83			0.28	0.779	0.16	0.68	0.498

Change from baseline scores are listed separately for the post-assessment session (immediately after the 4-week nasal spray administration period, T1) and the follow-up assessment (4 weeks after cessation of the nasal spray administration period, T2)

For all outcomes, except ASCQ secure and Attachment mother secure, negative change from baseline scores indicate pre-to-post-improvement

Values printed in bold indicate *p*-values < 0.05

*SRS-2* social responsiveness scale, *RBS-R* repetitive behavior scale-revised, *SCARED* screen for child anxiety related disorders, *ASCQ* attachment style classification questionnaire

All outcomes were assessed five times: (i) at baseline (T0), (ii) immediately after the 4-week double-blind nasal spray administration period (phase I—post, T1); (iii) at a follow-up session, 4 weeks after cessation of the double-blind nasal spray administration period (phase I—follow-up, T2); (iv) immediately after the 4-week single-blind nasal spray administration period (phase II—post, T3); and (v) at a follow-up session 4 weeks after cessation of the single-blind nasal spray administration period (phase II—follow-up, T4). Post-sessions were scheduled approximately 24 h after the last administration, follow-up sessions within 28 ± 7 days.

### Data analysis

Analyses were performed using a modified intention-to-treat approach that included all randomized participants who completed the baseline session and at least one post or follow-up session (Fig. 1B, **CONSORT diagram**). All statistics were executed with Statistica 14 (Tibco Software Inc.).

First, possible baseline differences on the questionnaires were assessed between randomized nasal spray groups, indicating no statistically significant differences (Table 1). Next, between-group differences in treatment responses of phase I (double-blind) on the primary and secondary outcome measures were assessed, by subjecting change from baseline scores of the post (T1) and follow-up (T2) sessions to independent sample *t*-tests. Cohen’s *d* effect sizes (change from baseline_OT_—change from baseline_PLACEBO_)/pooled SD) are also reported, where 0.2 is indicative of a small effect, 0.5 a medium effect and 0.8 a large effect. Additionally, single-sample *t*-tests were adopted to assess within-group changes (compared to baseline) in the OT and placebo group separately (Table 2 and Additional file 1: Table S6). Similar independent and single-sample *t*-tests were adopted to assess treatment responses of phase II (single-blind), although note that here changes in outcome measures were calculated relative to assessment session T2 (last session of phase I), i.e., allowing to examine treatment-induced changes, over and above changes induced in phase I (Additional file 1: Table S6 and S7).

Further, to assess whether the overall magnitude of treatment-induced changes at the last session of the trial (T4) (calculated as change from baseline T0, i.e., reflecting the total change over phases I and II) were reliable for individual participants (more than can be expected by measurement error), the Reliable Change Index (RCI) [34] was calculated, based on the test–retest reliability of the adopted Dutch parent-reported SRS scale (Cronbach’s alpha = 0.94) and corresponding standard error of measurement (SEM = baseline SD × SQRT(1—Cronbach’s alpha) = 5.19) using the formula: RCI = 1.96 × SQRT (2 × SEM × SEM) = 14.8. Change scores higher than the RCI-value (14.8) were considered reliable.

Finally, exploratory analyses were performed to investigate the potential influence of moderator variables on phase I treatment outcome. To do so, change from baseline scores were subjected to a mixed-effect model with ‘subject’ as random factor and ‘nasal spray’ (OT, placebo), ‘assessment session’ (T1 post, T2 follow-up) and the moderator variable included as fixed factors. Separate models were constructed to assess the modulating effect of concomitant psychosocial training (3 or more sessions per month, less than 3 sessions per month); medication use (present, not present; as listed in Table 1); biological sex (male, female); and parent-reported beliefs (OT, placebo).

## Results

### Double-blind phase (phase I)

No significant effect of ‘nasal spray’ was revealed on parent-reported social responsiveness (SRS-2), neither at the T1 assessment session, immediately after the 4-week nasal spray administration period (*p* = 0.839), nor at the 4-week follow-up session (T2, *p* = *0.6*26) (see Table 2 and Additional file 1: Table S6). Both groups displayed significant but similar pre-to-post-treatment improvements in social responsiveness (reduced SRS-2 scores) immediately after the nasal spray administration period (OT: *p* = 0.017; placebo: *p* = 0.009) and at the follow-up session (OT: *p* = 0.001; placebo: *p* = 0.017). A similar pattern of non-treatment-specific improvements was evident for the secondary outcomes (Table 2).

### Single-blind phase (phase II)

**Fig. 2:**
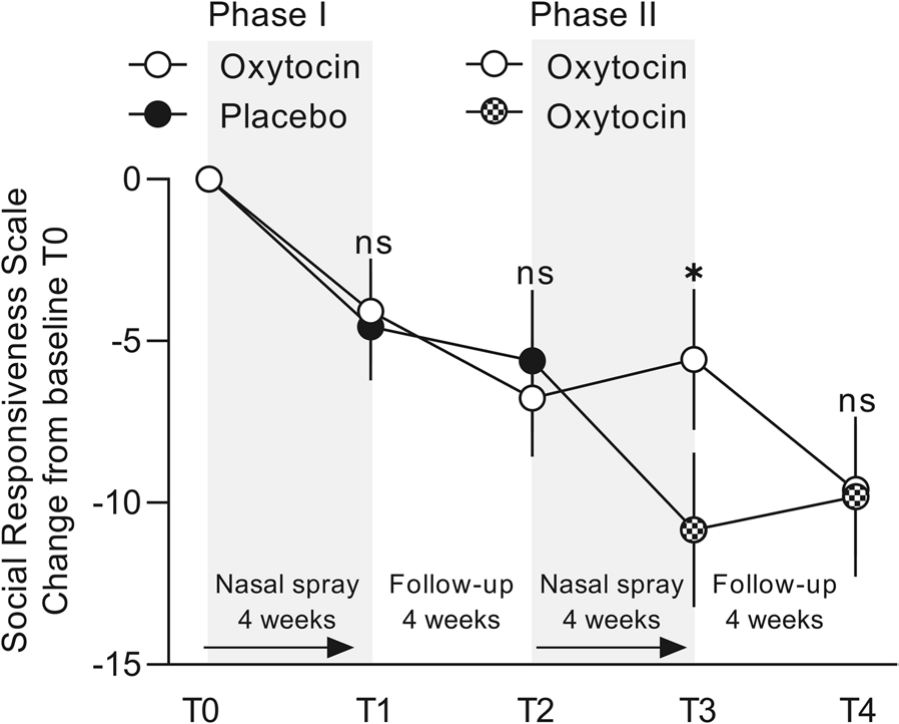
Effects of oxytocin nasal spray administration on social responsiveness. Visualization of changes from baseline in caregiver-reported social responsiveness (SRS-2 total raw scores) of the double-blind phase (phase I) and the single-blind phase (phase II), separately for each original nasal spray group (oxytocin-first, placebo-first) and assessment session (immediate post (T1 and T3)and four-week follow-up (T2 and T4)). Lower scores indicate improvement. Vertical bars denote ± standard errors

To examine whether the change from placebo to OT administration (in the placebo-first group) or a continuation of OT administration for another 4 weeks (in the OT-first group) may have induced differential changes in SRS-2 scores during phase II, change scores (from T2, the last session of phase I) were calculated and subjected to independent-sample t-tests with the between-subject factor ‘Phase I nasal spray’ (OT-first versus placebo-first). At assessment session T3 (immediately after the single-blind administration period), a significant effect of ‘Phase I nasal spray’ was evident, indicating that children who continued from the placebo nasal spray to the OT nasal spray showed stronger improvements in social responsiveness (*t*(73) = 2.91; *p* = 0.005), compared to children of the OT-first group, who already received a 4-week course of OT in phase I and received another 4-week course of OT administration in phase II (Fig. 2; Additional file 1: Table S6 for the raw scores). Indeed, only in the placebo-first group, significant improvements in SRS scores were evident (change from T2 to T3: *t*(38) = − 3.27; *p* = 0.002), whereas in children of the OT-first group, the extra 4-week course of OT administration did not yield further improvements in SRS scores (change from T2 to T3: *t*(37) = 0.86; *p* = 0.394).

At the T4 follow-up session, the difference between the OT-first and placebo-first groups was no longer significant (*t*(70) = 0.24; *p* = 0.808); here, a significant improvement in SRS scores was evident across both groups (change from T2 to T4: *t*(72) = − 2.72; *p* = 0.008). A similar pattern of improvements in the placebo-first group was evident for the secondary outcomes repetitive behaviors and anxiety symptoms (see Additional file 1: Table S7).

Accordingly, as seen in Fig. 2, at T4, the last session of the trial, both the OT-first (receiving a total of 8 weeks of OT nasal spray) and the placebo-first group (receiving a total of 4 weeks of OT nasal spray) displayed significant improvements in social responsiveness, when compared to their initial T0 baseline score, at the start of the trial (OT-first; pre-post-change: − 9.61 ± 12.18; *t*(35) = − 4.74; *p* < 0.001; placebo-first; pre-post-change: − 9.81 ± 14.83; *t*(35) = − 3.97; *p* < 0.001). In the OT-first group, 27 (out of 36: 75%) participants displayed a pre-to-post-improvement, and this change was identified to be reliable for 12 participants (higher than the Reliable Change Index: > 14.8). Similarly, also in the placebo-first group, 27 (out of 36: 75%) participants displayed a pre-to-post-improvement at the last session of the trial, which was reliable for 11 participants.

### Exploratory moderator analyses

**Fig. 3:**
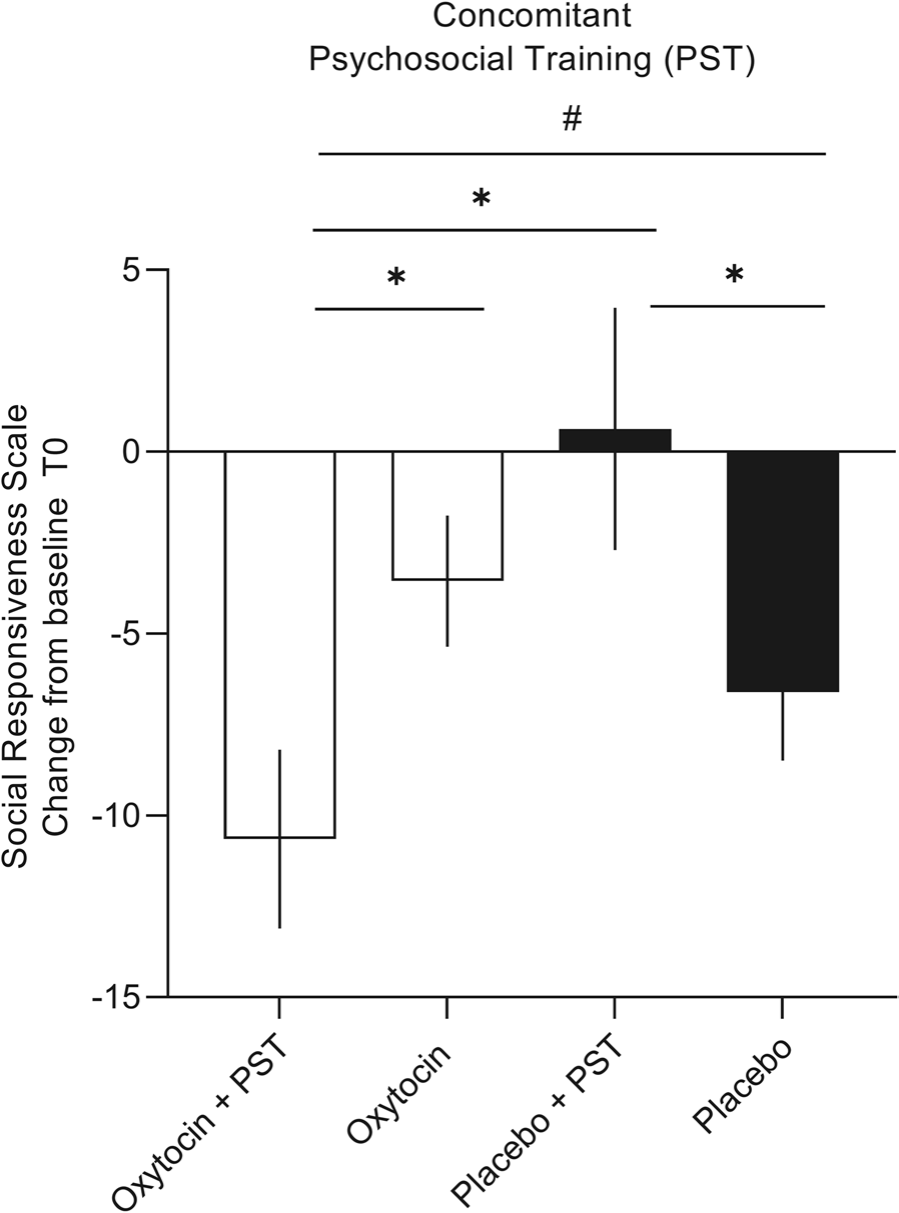
Change in treatment responses according to the presence of concomitant psychosocial training. Visualization of changes from baseline in parent-reported social responsiveness (SRS-2 raw total scores) of the double-blind phase (phase I), separately for children receiving only the oxytocin (*n* = 28) or placebo (*n* = 30) nasal spray and children receiving oxytocin (*n* = 10) or placebo (*n* = 8) nasal spray in combination with concomitant psychosocial trainings (pooled across the immediate post and four-week follow-up sessions of phase I). Lower scores indicate improvement. Vertical bars denote ± standard errors

For the moderator variable ‘psychosocial training’, a significant interaction with ‘nasal spray’ was identified (*F*(1,72) = 7.46; *p* = 0.007; *η*_*p*_^2^ = 0.09; Fig. 3), indicating that, across assessment sessions (T1 post, T2 follow-up), participants who received the OT nasal spray combined with a high intensity of psychosocial training (3 or more session per month) (n = 10) displayed greater benefits compared to children receiving the OT nasal spray alone or with a low intensity of psychosocial training (less than 3 sessions per month) (*n* = 28) (*p*_Bonferroni_ = 0.002). Notably, treatment responses of the placebo group were modulated in the opposite direction, indicating that reported changes in social responsiveness were most pronounced for children of the placebo group who received no or only low-intensity psychosocial training (*n* = 30), compared to children who received the placebo nasal spray with a high intensity of ongoing psychosocial trainings (*n* = 8) (*p*_Bonferroni_ = 0.004).

Direct comparisons showed that improvements in social responsiveness in the subgroup of children receiving the OT nasal spray in combination with psychosocial training (see Fig. 3) were more pronounced compared to improvements seen in children of the placebo group with (*p*_Bonferroni_ < 0.001) or without psychosocial trainings, albeit for the latter comparison, the effect was only significant at an uncorrected threshold (*p*_uncorrected_ = 0.035; *p*_Bonferroni_ = 0.21).

For all the other assessed moderator variables (concomitant medication use, sex or parent-reported beliefs), no significant main effects or interactions with nasal spray were identified (all, *p* > 0.14), indicating no significant moderation of treatment responses by these factors.

## Discussion

The current pediatric trial demonstrated no significant treatment-specific effects of a 4-week OT administration period on social responsiveness (SRS-2), nor on the secondary outcomes. Both the OT and the placebo groups displayed similar improvements, both immediately after the multiple-dose nasal spray administration and at the 4-week follow-up session. Notably, participants who were allocated to receive the placebo nasal spray during the first double-blind phase of the trial and continued to receive the active nasal spray during the second (single-blind) phase, displayed a significant improvement in social responsiveness, over and above the placebo-induced improvement noted in the first phase. Finally, albeit exploratory, moderator analyses preliminarily showed that children who received the OT nasal spray in combination with concomitant psychosocial training (three or more sessions per month) displayed a greater improvement in social responsiveness compared to children receiving the OT nasal spray alone or to children receiving the psychosocial training alone.

Results of earlier multiple-dose OT trials in children with ASD have been equivocal: some with beneficial outcomes [15, 16], others without significant effect [17–19]. While it is difficult to pinpoint the different factors contributing to variability in study results, several key differences in adopted dosing scheme, trial design, and participant demographics have been put forward as important moderators. Furthermore, the particular context in which the OT is administered is also increasingly put forward as a vital factor for understanding variability in treatment responses within and across studies. Initial single-dose administration studies already noted that acute effects of OT can be modulated by contextual factors, indicating, for instance, that OT-induced facilitation of cooperation and trust is most pronounced toward in-group members [35, 36]. Also, stress-reducing effects of OT were significantly augmented when accompanied by a supportive context (i.e., social support from a friend) [37]. Against this background, it has been theorized that OT may open a ‘window of opportunity’ to enhance prosocial behavior, but that its potential can only be fully realized when OT is administered within a (socially) stimulating context, such as effective concomitant behavioral interventions that can support social skill development and improve prosocial behavior [20, 22]. In line with this notion, exploratory assessments within our study revealed a significant synergetic modulation of treatment outcome related to the presence of concomitant psychosocial trainings/therapies during the course of the OT trial, indicating maximal efficacy in children receiving the OT nasal spray in combination with ongoing psychosocial trainings. Administration of OT as an adjunct to other therapeutic approaches has been explored before. For example, in a study including adults with a diagnosis of schizophrenia, 6-week social cognition training was combined with OT administration, yielding significant improvements in empathic accuracy [38]. Also, in male adults with a diagnosis of social anxiety disorder, OT administered as an adjunct to 4 sessions of public speaking-exposure therapy-induced significant improvements in mental representations of the self [39]. While preliminary, a recent 6-week OT administration study in which parents were stimulated to systematically engage with their child in a positive social interaction or play session in the first hour after spray administration, yielded positive treatment outcomes in 46 3- to 8-year-old children with ASD, both in terms of social improvements and repetitive behaviors [23]. Together, this work highlights the relevance of context and urges future clinical trials to further elucidate whether clinical efficacy can be augmented when OT administration is paired with targeted behavioral interventions that support similar states and (social) behaviors [22].

Further, in phase II of the trial it was observed that children who continued from placebo (in phase I) to the actual OT nasal spray (in phase II) showed a significant further improvement in social responsiveness over and above the substantial placebo-induced improvement noted in phase I. Trial designs in which a phase of blinded placebo intervention is administered before actual nasal spray allocation have been put forward as an effective method to control for placebo effects and to improve detection of ‘real’ therapeutic responses [15]. The current observation of a significant further improvement from a blinded placebo phase to the active nasal spray provides support to this notion.

Children who received the actual OT nasal spray in the first phase and continued to a second phase of active nasal spray administration did not show a further improvement immediately after the second nasal spray administration period, although further changes did become apparent at the follow-up session, 4 weeks after cessation of the second nasal spray administration period. It is noted indeed, that at the last follow-up session of the trial, the majority of children of both the OT-first group and the placebo-first group displayed (reliable) beneficial effects in social responsiveness, indicating that both an 8-week (with a 4-week break in the middle) or a continual 4-week OT administration period were similarly able to induce a significant beneficial outcome in the social domain. This observation adds to the field’s uncertainty regarding to-be-administered dosing schemas and durations. In multiple-dose OT trials with individuals with ASD, daily dosing ranged from 8 to 80 IU and durations from 4 continual days to 24 weeks, but strong empirical support for favoring one dosing scheme over another is currently lacking. Some earlier single-dose trials suggested dose–response curves to exhibit U-shaped forms [40, 41], a notion that is supported by a recent chronic 4-week OT administration trial in ASD, identifying a daily total dose of 6 IU of TTA-121 (a new formulation of intranasal OT spray) to be the most efficacious one, compared to a lower (3 IU) or higher (10 IU) daily dose [42]. Furthermore, in terms of dosing scheme, recent work showed that intermittent (every other day) administration may be therapeutically more efficient than continual administration to obtain anxiolytic effects [43]. These observations were attributed to reflect a desensitization of the endogenous oxytocinergic system upon too high concentrations and/or too high frequencies of exogenous OT administration. The current observation that a single 4-week course yields similar immediate effects as a twice 4-week course therefore reinforces the notion that longer durations of nasal spray administration periods do not necessarily facilitate higher treatment responses. Similarly, in a recent large-scale trial administering OT over a 24-week period, it was noted that the long duration might have attenuated initial early responses to OT [19]. In light of these observations, future trials should be directed at identifying the optimal dosing, administration length, and intervals of intranasal OT administration.

## Limitations

While the study provides novel insights into the effects of OT administration in school-aged children with ASD, the following limitations are noted. First, the current study included a relatively strict age range of pre-pubertal, school-aged children with ASD limiting generalizability to other age ranges. Future studies, using larger cohorts, are warranted to further explore the identified effects, also including understudied populations within the ASD spectrum, such as children with co-occurring mild-to-severe intellectual disabilities (i.e., the most common co-occurring condition with a prevalence ranging between 30 and 40%) [2, 44]. Also, considering the aforementioned uncertainty regarding dosing schemes, it is uncertain whether the identified effects will replicate using differential dosing schemes/durations. Further, while the SRS questionnaire has been frequently adopted as a social skill endpoint in clinical trials [45], including recent OT trials [16, 23], it constitutes a measure of parental observations, not without limitations due to biases of parental reports and prior beliefs and expectations. Future trials should preferentially also include clinician-rated observational scales specifically developed for measuring treatment-related change in socio-communicative behaviors (e.g. the Brief Observation of Social Communication Change [46]). Finally, considering the identified moderating effect of concomitant psychosocial trainings, future studies are urged to monitor, standardize and experimentally manipulate and implement concurrent behavioral interventions to elucidate its potential for modulating treatment efficacy.

## Conclusions

To conclude, while the current study showed no overall treatment-specific improvements, exploratory moderator effects were identified, providing preliminary evidence that clinical efficacy can be augmented when OT administration is paired with targeted concurrent behavioral interventions.

## Supplementary Information

**Additional file1**. In this file, more detailed information is provided regarding supplementary methods and results. The file consists of a supplementary method section and seven supplementary tables, supporting the content of the main manuscript.

## Data Availability

The data that support the findings of this study are available on request from the corresponding author, KA. The data are not publicly available due to privacy restrictions.

## Abbreviations

ADOS-2: Autism Diagnostic Observation Schedule
ASCQ: Attachment Style Classification Questionnaire
ASD: Autism spectrum disorder
CONSORT: Consolidated Standards of Reporting Trials
DSM: Diagnostic and Statistical Manual of Mental Disorders
IQ: Intelligence quotient
IU: International units
OT: Oxytocin
RBS-R: Repetitive behavior scale-revised
RCI: Reliable change index
RCT: Randomized controlled trial
SCARED-NL: Screen for Child Anxiety Related Emotional Disorders (Dutch Version)
SRS-2: Social Responsiveness Scale
