# Supplementary material for: Effects of multiple-dose intranasal oxytocin administration on social responsiveness in children with autism: a randomized, placebo-controlled trial
Source: Mol Autism. 2023 Apr 20;14:16. doi: 10.1186/s13229-023-00546-5 (PMC10117268; doi:10.1186/s13229-023-00546-5)
Supplement: Supplementary file 1 — Additional file1. In this file, more detailed information is provided regarding supplementary methods and results. The file consists of a supplementary method section and seven supplementary tables, supporting the content of the main manuscript. [file 13229_2023_546_MOESM1_ESM.pdf]

Supplementary Information

Effects of multiple-dose intranasal oxytocin administration on social responsiveness in children with autism: A randomized, placebo-controlled trial

Authors

Nicky Daniels, Matthijs Moerkerke, Jean Steyaert, Annelies Bamps, Edward Debbaut, Jellina Prinsen, Tiffany Tang, Stephanie Van de Donck, Bart Boets, Kaat Alaerts

Supplementary Methods

Rationale for adopted trial design. As outlined, all outcomes were assessed five times: (i) at baseline (T0), (ii) immediately after the four-week double-blind nasal spray administration period (phase I – post, T1); (iii) at a follow-up session, four weeks after cessation of the double-blind nasal spray administration period (phase I - follow-up, T2); (iv) immediately after the four-week single-blind nasal spray administration period (phase II – post, T3); and (v) at a follow-up session four weeks after cessation of the single-blind nasal spray administration period (phase II - follow-up, T4) (see **Figure 1A** for a visualization of the trial design).

Generally, the double-blind, parallel trial design of phase I (with the T1 post and T2 follow-up) was similar to our prior OT trial with adults [1] allowing to examine the possibility of retention effects in the current pediatric sample.

After completion of the T2 follow-up session of Phase I, participants were further enrolled in the second 'single-blind' phase (phase II), during which all participants received the OT nasal spray for a course of four weeks. The cross-over of participants of the placebo-first group to the oxytocin nasal spray allowed all participants to receive the actual OT nasal spray during their trial participation and therefore, allowed obtaining assessments of OT-induced changes from all children participating in the trial. Importantly, obtaining outcome assessments from a larger total number of children receiving the OT nasal spray allowed performing more thorough examinations of possible variations in treatment responses due to moderator variables (see results).

Further, children who already received OT in the double-blind phase, were also given another four-week course of OT administration during the subsequent single-blind phase. A cross-over of this group to the placebo nasal spray was deemed suboptimal, considering recent notions of long-lasting retention effects upon multiple-dose oxytocin administration [1], which may have interfered with a true assessment of placebo nasal spray administration. Furthermore, allowing the oxytocin-first group to receive an additional four-week course of OT allowed an exploratory examination of potential augmenting effects upon receiving the nasal spray for a longer duration.

COVID-19 related impact. As indicated in the initial EudraCT trial registration, participants were recruited to participate in a larger project, also including physiological, neural, neurophysiological and biological assessments, for which a total sample size of 60 participants was planned (30 in each treatment arm). However, due to COVID-19 related health restrictions, all physiological, neural and neurophysiological data collections were temporarily halted, resulting in extensive loss of treatment follow-up data on these neural outcomes. Fortunately, for all participants already enrolled or scheduled to initiate the nasal spray administration, the collection of the behavioral data through questionnaires could still be continued during the COVID-19 lock-down periods. Nonetheless, to account for the extensive data loss on the neurophysiological assessments, and upon approval from the Ethical committee, recruitment was extended after the COVID-19 lock-downs, allowing to still obtain the initially planned sample size of 30 participants in each treatment arm on the neurophysiological assessments. As a result, the total sample size of participants in the trial (and for whom behavioral collections were performed) was increased from 60 to 80.

Reference: [1] Bernaerts S, Boets B, Bosmans G, Steyaert J, Alaerts K. Behavioral effects of multiple-dose oxytocin treatment in autism: A randomized, placebo-controlled trial with long-term follow-up. Mol Autism. 2020;11:6.

Supplementary Table 1

Full list of inclusion and exclusion criteria.

Inclusion criteria	Exclusion criteria
Diagnosed with ASD by a multidisciplinary team of experienced clinicians as defined by the DSM-IV-TR or DSM-5-TR criteria (Diagnostic and Statistical Manual of Mental Disorders).	History of any neurological disorder (stroke, concussion, epilepsy etc).
Age-range of 8 to 12 years old.	Significant hearing or vision impairments.
Premenstrual girls (girls with onset of menstruation during the course of the trial are allowed to continue the treatment).	Active medical problems: unstable seizures, significant physical illness (e.g., serious liver, renal, or cardiac pathology).
Intelligence Quotient above 70 (either full-scaled IQ, verbal IQ or performance IQ).	Regular nasal obstruction or nosebleeds.
Dutch native speaker.	Subjects who have had previous chronic treatment with oxytocin.
Stable background treatment during four weeks prior to screening.	Participation in another Clinical Trial.
No planned changes in psychosocial interventions during the trial.	Known hypersensitivity to active substance or excipients in nasal sprays.
	(Significant) change in background treatments.
	For MR assessment: any contraindication to MRI research (pacemaker, implanted defibrillator, ear implant / a cochlear implant, insulin or implanted pump, a neurostimulator or VP shunt, any metallic object in the eyes (metallic fragments)*.

*As indicated in the registration at the European Clinical Trial Registry (EudraCT 2018-000769-35), behavioral data collections were part of a larger project assessing neurophysiology and biological outcomes.

Supplementary Table 2

Detailed information on medication use and co-occurring conditions for participants of the oxytocin and placebo treatment groups.

Current psychoactive medication use was defined as use within four weeks before study enrollment. Co-occurring conditions were screened through parent-report (with the explicit mentioning of examples in the screening interview including e.g., ADHD, depression, dyscalculia, dyslexia).

All participants had a stable background treatment for at least four weeks prior to the treatment allocation and changes in medication regime were screened and logged.

	Oxytocin (n = 38)		Placebo (n = 39)		Pearson Chi square	*p*-value
	no.	(%)	no.	(%)		
**Psychoactive Medication**						
Antianginal agents	1	(2.5%)	0	(0%)	1.04	0.31
Anticholinergic agent	3	(7.5%)	0	(0%)	3.20	0.07
Anti-depressants	3	(7.5%)	1	(2.5%)	1.11	0.29
Antipsychotics	6	(15%)	7	(17.5%)	0.06	0.80
Sleep Aids	6	(15%)	12	(30%)	2.41	0.12
Stimulants	11	(27.5%)	9	(22.5%)	0.34	0.56
**Other Medication**						
Allergy and asthma medications	3	(7.5%)	1	(2.5%)	1.11	0.29
Gastrointestinal medications	0	(0%)	2	(5%)	2.00	0.16
Nutritional Supplements	2	(5%)	4	(10%)	0.67	0.41
Statins	1	(2.5%)	0	(0%)	1.04	0.31
**Co-occurring conditions**						
ADHD	11	(28.9%)	10	(25.6%)	0.11	0.74
DCD	3	(7.5%)	1	(2.5%)	1.11	0.29
Dyslexia	2	(5%)	5	(12.5%)	1.33	0.25
Dysorthography	0	(0%)	1	(2.5%)	0.99	0.32
OCD	1	(2.5%)	0	(0%)	1.04	0.31

ADHD, Attention Deficit Hyperactivity Disorder; DCD, Developmental Coordination Disorder; OCD, Obsessive-Compulsive Disorder.

Supplementary Table 3

Side effect screening.

Participants (with the help of their parents) were asked to administer the nasal spray (oxytocin or placebo) daily for four consecutive weeks in phase I (double-blind phase) and another four weeks in phase II (single-blind extension phase). At the end of each week, parents were asked to report whether their child presented any of the listed (or other) side effects and to indicate the severity of the side effect (mild, moderate, or severe). Safety analyses included all participants that received the allocated intervention.

Panel A lists the proportion of oxytocin or placebo participants (%) that reported any mild, moderate or severe side effects (averaged across effects). P-values correspond to hypothesis tests for difference in proportions between the two treatment groups. Data printed in bold show p-values equal to or larger than 0.05.

Panel B lists, separately for each side effect, the proportion of oxytocin (OT) or placebo (PL) participants that reported the side effect (averaged across severity level: mild, moderate, severe).

Panel C lists any other incidental adverse event, spontaneously reported by the parents.

While on average, no group differences were evident in the total proportion of reported side effects, a group differences was observed in the proportion of participants who reported moderate side effects in the last week of administration, indicating that the participants of the oxytocin group reported a slightly higher number of moderate side effects during that week.

When examined separately for each side effect, a higher proportion of participants of the oxytocin group reported to experience a ‘headache’ or ‘abdominal/stomach pain’ during the third week of the phase I treatment. Also a higher proportion of participants of the placebo group were noted to experience a ‘sore throat’ and to feel ‘more confident’, compared to the oxytocin group, but only during the first week of the phase I treatment.

In the single-blind phase (phase II), no significant group differences were revealed, either in the total proportion of reported side effects (**Panel A**) or separately for each side effect (**Panel B**).

Panel A

Phase I (double-blind)

	**Oxytocin (%)**			**Placebo (%)**			**Group difference (*p*-value)**		
	**Mild**	**Moderate**	**Severe**	**Mild**	**Moderate**	**Severe**	**Mild**	**Moderate**	**Severe**
**Week 1**	30.00	27.50	2.50	32.50	15.00	0.00	0.81	0.17	0.31
**Week 2**	37.50	25.00	2.50	22.50	17.50	0.00	0.14	0.41	0.31
**Week 3**	22.50	20.00	5.00	22.50	10.00	5.00	1.00	0.21	1.00
**Week 4**	35.00	20.00	2.50	30.00	5.00	2.50	0.63	**0.04**	1.00
**Across weeks**	31.25	23.13	3.13	26.88	11.88	1.88	0.67	0.19	0.72

Phase II (single-blind)

	**Oxytocin$_{first}$ (%)**			**Placebo$_{first}$ (%)**			**Group difference (*p*-value)**		
	**Mild**	**Moderate**	**Severe**	**Mild**	**Moderate**	**Severe**	**Mild**	**Moderate**	**Severe**
**Week 1**	34.21	21.05	7.89	23.08	23.08	10.26	0.28	0.83	0.72
**Week 2**	28.95	23.68	13.16	17.95	17.95	2.56	0.25	0.54	0.08
**Week 3**	23.68	13.16	5.26	17.95	7.69	2.56	0.54	0.43	0.54
**Week 4**	34.21	26.32	2.63	35.90	28.21	0.00	0.88	0.85	0.31
**Across weeks**	30.26	21.05	7.24	23.72	19.23	3.85	0.52	0.84	0.51

Panel B

Phase I (double-blind)
(OT *n* = 40; PL *n* = 40)

	**Week 1**			**Week 2**			**Week 3**			**Week 4**		
	**OT (%)**	**PL (%)**	***p***	**OT (%)**	**PL (%)**	***p***	**OT (%)**	**PL (%)**	***p***	**OT (%)**	**PL (%)**	***p***
Headache	10.00	12.50	0.72	7.50	7.50	1.00	12.50	0.00	**0.02**	5.00	2.50	0.56
Drowsiness	2.50	2.50	1.00	7.50	5.00	0.64	7.50	0.00	0.08	2.50	0.00	0.31
Dizziness	7.50	2.50	0.30	2.50	2.50	1.00	7.50	0.00	0.08	2.50	0.00	0.31
Fainting	0.00	0.00	1.00	0.00	0.00	1.00	2.50	0.00	0.31	0.00	0.00	1.00
Changes in heart rate or palpitations	0.00	0.00	1.00	2.50	0.00	0.31	2.50	0.00	0.31	0.00	0.00	1.00
Shortness of breath	0.00	0.00	1.00	0.00	0.00	1.00	0.00	2.50	0.31	0.00	0.00	1.00
Fever	0.00	2.50	0.31	0.00	0.00	1.00	0.00	0.00	1.00	2.50	0.00	0.31
Sore throat	0.00	12.50	**0.02**	5.00	7.50	0.64	5.00	0.00	0.15	0.00	0.00	1.00
Dry throat/dry mouth	0.00	5.00	0.15	0.00	0.00	1.00	2.50	0.00	0.31	5.00	0.00	0.15
Hoarseness	0.00	5.00	0.15	0.00	2.50	0.31	0.00	0.00	1.00	2.50	0.00	0.31
Coughing	5.00	5.00	1.00	2.50	2.50	1.00	2.50	0.00	0.31	5.00	0.00	0.15
Coughing up mucus	0.00	0.00	1.00	0.00	0.00	1.00	0.00	0.00	1.00	0.00	0.00	1.00
Congested nose	10.00	7.50	0.69	5.00	10.00	0.40	10.00	5.00	0.40	5.00	5.00	1.00
Sneezing	2.50	2.50	1.00	0.00	2.50	0.31	7.50	2.50	0.30	7.50	2.50	0.30
Nasal irritation	5.00	5.00	1.00	5.00	0.00	0.15	7.50	2.50	0.30	2.50	0.00	0.31
Runny nose	2.50	2.50	1.00	2.50	2.50	1.00	2.50	0.00	0.31	7.50	0.00	0.08
Burning sensation in nose and/or ears	5.00	0.00	0.15	5.00	0.00	0.15	5.00	0.00	0.15	2.50	0.00	0.31
Sensitive to fragrances	0.00	0.00	1.00	0.00	0.00	1.00	0.00	0.00	1.00	2.50	0.00	0.31
Watery eyes	0.00	0.00	1.00	2.50	0.00	0.31	0.00	0.00	1.00	2.50	0.00	0.31

Nausea and/or vomiting	2.50	7.50	0.30	5.00	2.50	0.56	2.50	0.00	0.31	0.00	0.00	1.00
Abdominal or stomach pain	12.50	10.00	0.72	15.00	7.50	0.29	12.50	0.00	**0.02**	7.50	7.50	1.00
Decreased appetite	2.50	5.00	0.56	2.50	2.50	1.00	5.00	0.00	0.15	2.50	0.00	0.31
Hungry or increased appetite	0.00	2.50	0.31	0.00	2.50	0.31	2.50	0.00	0.31	0.00	0.00	1.00
Constipation	7.50	2.50	0.30	5.00	0.00	0.15	7.50	0.00	0.08	7.50	2.50	0.30
Diarrhea	0.00	2.50	0.31	2.50	2.50	1.00	2.50	0.00	0.31	0.00	0.00	1.00
Muscle pain/cramps	5.00	0.00	0.15	5.00	0.00	0.15	5.00	0.00	0.15	5.00	2.50	0.56
Skin rash	2.50	0.00	0.31	2.50	0.00	0.31	0.00	0.00	1.00	5.00	0.00	0.15
Increased fluid intake	0.00	0.00	1.00	0.00	0.00	1.00	2.50	0.00	0.31	0.00	0.00	1.00
Water retention/bloating	0.00	0.00	1.00	2.50	0.00	0.31	0.00	0.00	1.00	2.50	0.00	0.31
Insomnia/sleep difficulties	5.00	0.00	0.15	5.00	5.00	1.00	5.00	0.00	0.15	0.00	5.00	0.15
Nightmares	2.50	0.00	0.31	0.00	0.00	1.00	0.00	0.00	1.00	0.00	0.00	1.00
Staring/daydreams	0.00	0.00	1.00	2.50	0.00	0.31	0.00	0.00	1.00	2.50	0.00	0.31
Anaphylaxis	0.00	0.00	1.00	0.00	0.00	1.00	0.00	0.00	1.00	0.00	0.00	1.00
Changes in perception of the tongue	0.00	0.00	1.00	0.00	0.00	1.00	0.00	0.00	1.00	0.00	0.00	1.00
Back pain	0.00	0.00	1.00	0.00	0.00	1.00	0.00	0.00	1.00	0.00	0.00	1.00
Bed wetting	0.00	2.50	0.31	0.00	5.00	0.15	0.00	0.00	1.00	0.00	0.00	1.00
Weight gain	0.00	0.00	1.00	0.00	0.00	1.00	0.00	0.00	1.00	0.00	0.00	1.00
Sweating	0.00	0.00	1.00	2.50	0.00	0.31	0.00	0.00	1.00	0.00	0.00	1.00
Blurred vision	0.00	0.00	1.00	0.00	0.00	1.00	0.00	0.00	1.00	0.00	0.00	1.00
Less talk to others	0.00	0.00	1.00	0.00	2.50	0.31	2.50	0.00	0.31	2.50	2.50	1.00
Uninterested in others	0.00	0.00	1.00	0.00	2.50	0.31	0.00	0.00	1.00	2.50	0.00	0.31
Persistent thoughts and/or feelings	2.50	7.50	0.30	0.00	5.00	0.15	2.50	2.50	1.00	0.00	2.50	0.31
Development of repetitive behavior	0.00	0.00	1.00	2.50	0.00	0.31	0.00	2.50	0.31	0.00	0.00	1.00
Increase in repetitive behavior	2.50	0.00	0.31	2.50	0.00	0.31	0.00	2.50	0.31	0.00	0.00	1.00
Nail biting	0.00	0.00	1.00	0.00	0.00	1.00	0.00	0.00	1.00	2.50	0.00	0.31
Irritability or Anger	0.00	7.50	0.08	2.50	7.50	0.30	7.50	5.00	0.64	10.00	7.50	0.69
Sad	2.50	0.00	0.31	0.00	7.50	0.08	2.50	5.00	0.56	5.00	5.00	1.00
Prone to crying or more emotional	2.50	5.00	0.56	2.50	12.50	0.09	7.50	12.50	0.46	5.00	7.50	0.64
Anxious, worried or discomfort	0.00	0.00	1.00	5.00	0.00	0.15	5.00	2.50	0.56	2.50	2.50	1.00
Happy or satisfied	10.00	25.00	0.08	7.50	15.00	0.29	17.50	15.00	0.76	7.50	12.50	0.46
Euphoric or unusually happy	5.00	10.00	0.40	7.50	7.50	1.00	7.50	5.00	0.64	10.00	2.50	0.17
Calm, relaxed or comfortable	10.00	25.00	0.08	15.00	17.50	0.76	12.50	22.50	0.24	7.50	10.00	0.69
More focused	0.00	2.50	0.31	0.00	5.00	0.15	2.50	2.50	1.00	0.00	2.50	0.31
More confidence	0.00	12.50	**0.02**	7.50	7.50	1.00	7.50	5.00	0.64	5.00	7.50	0.64

	**Phase II (single-blind) (OT_{first} *n* = 38; PL_{first} *n* = 39)**											
	**Week 1**			**Week 2**			**Week 3**			**Week 4**		
	**OT_{first} (%)**	**PL_{first} (%)**	***p***	**OT_{first} (%)**	**PL_{first} (%)**	***p***	**OT_{first} (%)**	**PL_{first} (%)**	***p***	**OT_{first} (%)**	**PL_{first} (%)**	***P***
Headache	5.26	10.26	0.41	2.63	7.69	0.32	10.53	5.13	0.38	5.26	2.56	0.54
Drowsiness	2.63	0.00	0.31	2.63	2.56	0.99	0.00	0.00	1.00	0.00	0.00	1.00
Dizziness	0.00	2.56	0.32	0.00	2.56	0.32	0.00	0.00	1.00	0.00	0.00	1.00
Fainting	0.00	0.00	1.00	0.00	0.00	1.00	0.00	0.00	1.00	0.00	0.00	1.00
Changes in heart rate or palpitations	0.00	0.00	1.00	0.00	0.00	1.00	0.00	0.00	1.00	5.26	0.00	0.15
Shortness of breath	0.00	0.00	1.00	0.00	0.00	1.00	0.00	2.56	0.32	0.00	0.00	1.00

Fever	0.00	2.56	0.32	0.00	2.56	0.32	0.00	0.00	1.00	0.00	2.56	0.32
Sore throat	2.63	5.13	0.57	2.63	7.69	0.32	5.26	5.13	0.98	5.26	0.00	0.15
Dry throat/dry mouth	0.00	2.56	0.32	7.89	2.56	0.29	5.26	2.56	0.54	5.26	2.56	0.54
Hoarseness	0.00	0.00	1.00	2.63	0.00	0.31	2.63	2.56	0.99	2.63	0.00	0.31
Coughing	2.63	2.56	0.99	2.63	0.00	0.31	2.63	2.56	0.99	5.26	0.00	0.15
Coughing up mucus	0.00	0.00	1.00	2.63	0.00	0.31	2.63	0.00	0.31	0.00	0.00	1.00
Congested nose	2.63	5.13	0.57	5.26	5.13	0.98	5.26	7.69	0.67	7.89	0.00	0.07
Sneezing	0.00	0.00	1.00	0.00	2.56	0.32	0.00	2.56	0.32	2.63	0.00	0.31
Nasal irritation	2.63	5.13	0.57	0.00	0.00	1.00	5.26	2.56	0.54	7.89	0.00	0.07
Runny nose	2.63	0.00	0.31	0.00	2.56	0.32	2.63	5.13	0.57	0.00	5.13	0.16
Burning sensation in nose and/or ears	0.00	2.56	0.32	0.00	0.00	1.00	2.63	0.00	0.31	0.00	0.00	1.00
Sensitive to fragrances	0.00	0.00	1.00	2.63	2.56	0.99	2.63	0.00	0.31	2.63	0.00	0.31
Watery eyes	0.00	0.00	1.00	0.00	0.00	1.00	2.63	0.00	0.31	2.63	0.00	0.31
Nausea and/or vomiting	2.63	0.00	0.31	5.26	0.00	0.15	2.63	2.56	0.99	2.63	0.00	0.31
Abdominal or stomach pain	5.26	2.56	0.54	13.16	7.69	0.43	5.26	2.56	0.54	2.63	2.56	0.99
Decreased appetite	2.63	0.00	0.31	7.89	7.69	0.97	2.63	5.13	0.57	2.63	2.56	0.99
Hungry or increased appetite	0.00	0.00	1.00	2.63	2.56	0.99	0.00	0.00	1.00	0.00	0.00	1.00
Constipation	0.00	0.00	1.00	2.63	0.00	0.31	0.00	0.00	1.00	0.00	0.00	1.00
Diarrhea	5.26	0.00	0.15	5.26	0.00	0.15	0.00	0.00	1.00	0.00	0.00	1.00
Muscle pain/cramps	7.89	0.00	0.07	2.63	5.13	0.57	2.63	0.00	0.31	5.26	0.00	0.15
Skin rash	2.63	0.00	0.31	5.26	0.00	0.15	2.63	0.00	0.31	0.00	2.56	0.32
Increased fluid intake	0.00	2.56	0.32	2.63	0.00	0.31	5.26	2.56	0.54	2.63	0.00	0.31
Water retention/bloating	0.00	0.00	1.00	0.00	0.00	1.00	0.00	0.00	1.00	0.00	0.00	1.00
Insomnia/sleep difficulties	0.00	0.00	1.00	0.00	2.56	0.32	2.63	2.56	0.99	5.26	2.56	0.54
Nightmares	2.63	0.00	0.31	2.63	0.00	0.31	0.00	0.00	1.00	0.00	0.00	1.00
Staring/daydreams	2.63	0.00	0.31	2.63	2.56	0.99	0.00	0.00	1.00	0.00	0.00	1.00
Anaphylaxis	0.00	0.00	1.00	0.00	0.00	1.00	0.00	0.00	1.00	0.00	0.00	1.00
Changes in perception of the tongue	0.00	0.00	1.00	0.00	0.00	1.00	0.00	0.00	1.00	0.00	0.00	1.00
Back pain	0.00	0.00	1.00	0.00	0.00	1.00	0.00	0.00	1.00	0.00	0.00	1.00
Bed wetting	0.00	0.00	1.00	2.63	0.00	0.31	0.00	0.00	1.00	0.00	0.00	1.00
Weight gain	0.00	0.00	1.00	0.00	2.56	0.32	0.00	0.00	1.00	0.00	0.00	1.00
Sweating	0.00	0.00	1.00	2.63	0.00	0.31	2.63	2.56	0.99	2.63	2.56	0.99
Blurred vision	0.00	0.00	1.00	0.00	0.00	1.00	0.00	0.00	1.00	0.00	0.00	1.00
Less talk to others	0.00	0.00	1.00	0.00	7.69	0.08	0.00	2.56	0.32	2.63	2.56	0.99
Uninterested in others	0.00	0.00	1.00	0.00	2.56	0.32	2.63	0.00	0.31	2.63	0.00	0.31
Persistent thoughts and/or feelings	0.00	2.56	0.32	2.63	5.13	0.57	2.63	0.00	0.31	5.26	0.00	0.15
Development of repetitive behavior	5.26	0.00	0.15	0.00	0.00	1.00	0.00	2.56	0.32	0.00	0.00	1.00
Increase in repetitive behavior	0.00	0.00	1.00	2.63	2.56	0.99	0.00	0.00	1.00	0.00	0.00	1.00
Nail biting	2.63	0.00	0.31	2.63	0.00	0.31	0.00	0.00	1.00	0.00	2.56	0.32
Irritability or Anger	2.63	2.56	0.99	7.89	10.26	0.72	5.26	5.13	0.98	5.26	0.00	0.15
Sad	2.63	0.00	0.31	7.89	5.13	0.62	2.63	0.00	0.31	0.00	0.00	1.00
Prone to crying or more emotional	2.63	2.56	0.99	13.16	2.56	0.08	7.89	0.00	0.07	2.63	5.13	0.57
Anxious, worried or discomfort	2.63	0.00	0.31	5.26	2.56	0.54	5.26	0.00	0.15	2.63	0.00	0.31
Happy or satisfied	21.05	12.82	0.33	15.79	12.82	0.71	7.89	7.69	0.97	5.26	7.69	0.67
Euphoric or unusually happy	5.26	2.56	0.54	5.26	0.00	0.15	2.63	2.56	0.99	2.63	0.00	0.31

Calm, relaxed or comfortable	18.42	15.38	0.72	13.16	12.82	0.96	10.53	10.26	0.97	7.89	12.82	0.48
More focused	5.26	0.00	0.15	5.26	5.13	0.98	0.00	5.13	0.16	0.00	0.00	1.00
More confidence	7.89	5.13	0.62	7.89	5.13	0.62	0.00	7.69	0.08	0.00	7.69	0.08

**Panel C**	**Phase I (double-blind)**								**Phase II (single-blind)**							
**Number of other incidental events in spontaneous reports**	**Week 1**		**Week 2**		**Week 3**		**Week 4**		**Week 1**		**Week 2**		**Week 3**		**Week 4**	
	**OT**	**PL**	**OT**	**PL**	**OT**	**PL**	**OT**	**PL**	OT_{first}	PL_{first}	OT_{first}	PL_{first}	OT_{first}	PL_{first}	OT_{first}	PL_{first}
Nose bleeding	0	0	0	1	0	0	0	1	0	0	0	1	0	0	0	0
Fracture	0	0	0	0	0	0	0	0	0	0	0	0	0	0	0	1
Earache	0	0	1	0	0	0	0	0	0	0	0	0	0	0	0	0
Inappropriate Affect	0	1	0	0	0	1	0	1	0	1	0	1	1	0	1	1
Mood swings	0	0	0	0	0	0	1	0	0	0	0	0	0	0	0	0
Less focussed	1	0	0	0	0	0	1	0	3	0	2	0	0	0	0	0
Fecal incontinence	0	0	0	0	0	0	1	0	0	0	0	0	0	0	0	0
Feeling cold	0	1	0	0	0	0	0	0	0	0	0	0	0	0	0	0

Supplementary Table 4

Daily screenings of changes in affect and arousal.

Both participants and their parents completed a structured daily diary for four consecutive weeks in phase I (double-blind phase) and another four weeks in phase II (single-blind extension phase). Once daily, parents were asked to complete two 9-point Manikin rating scales, rating their child's perceived arousal (1= calm, 9= excited) and valence (1= feeling pleasant/happy, 9= feeling unpleasant/unhappy; [1]). Children also completed a self-report of the arousal and valence scale, twice-daily, once at noon and once in the evening.

To examine treatment-related differences in the daily screenings, weekly averages were calculated for each rating and subjected to a general linear model with the within-subject factor 'week' (week 1-4) and the between-subject factors 'treatment' (oxytocin, placebo).

None of the scales yielded a significant main effect of 'treatment' **across weeks** (all, $p > .05$), indicating no overall significant group differences in ratings of valence or arousal, either by the parents or by the child, both in phase I and phase II.

Closer analysis of treatment effects **separately for each week** revealed a significant group difference in child-ratings of valence (in the evening), indicating higher feelings of 'unpleasantness' in the oxytocin, compared to the placebo group during the second week of the phase I treatment. Also in the first week of phase II, a similar effect was noted, indicating that children who crossed over from the placebo treatment (in phase I) to the oxytocin treatment (in phase II) (placebo-first group) reported slightly higher feelings of 'unpleasantness', compared to children who were receiving their second course of oxytocin treatment (oxytocin-first group).

In the table below, weekly averages (and average responses across the four weeks) are reported separately for each treatment group (oxytocin, placebo) and phase (phase I and II). P-values correspond to independent-sample t-tests (or F-tests) assessing between-group differences in ratings of arousal and valence. Data printed in bold show p-values equal to or larger than 0.05.

Notably, for several of the scales, the general linear model also revealed a main effect of 'week', indicating that across treatment groups, both parents and children reported improvements in reports of valence (more pleasant) and arousal (more calm) from the first to the last week of the trial, particularly in phase II, when all children received the 'actual' oxytocin treatment (phase II, parent-reported valence:

$F(3,216) = 4.14$; $p = .007$; child-reported valence (noon & evening): $F(3,213) > 3.09$; $p < .028$; child-reported arousal (noon & evening) $F(3,213) > 6.88$; $p < .001$). Also in phase I, an overall effect of 'week' was evident in terms of child-reported valence (only at noon) ($F(3,195) = 5.62$; $p = .001$).

In addition to the ratings of arousal and valence, parents were also asked to indicate how they experienced the interaction with their child while completing the daily diary together, using a 5-point scale (1 = unpleasant/difficult; 5 = pleasant/easy). Generally, child-parent interactions while completing the daily diaries were experienced to be overall 'pleasant/easy', with slightly more pleasant experiences of the child-parent interaction in the oxytocin group, compared to the placebo group in phase I of the trial (oxytocin (n = 38): 4.32 ± 0.47; placebo (n = 36): 4.06 ± 0.58; $t(72) = 2.26$, $p = .027$). Differences in the experiences of the child-parent interaction were no longer evident in phase II of the trial, when all children received the actual oxytocin treatment (oxytocin-first (n = 36): 4.14 ± 0.73; placebo-first (n = 37): 3.84 ± 0.84; $t(71) = 1.62$, $p = .11$). Generally, these overall positive ratings provide an indication that the completion of the daily diaries was well-tolerated, both by the children and their parents.

Reference: [1] Bradley MM, Lang PJ. Measuring emotion: the Self-Assessment Manikin and the Semantic Differential. J. Behav. Ther. Exp. Psychiatry. 1994;25:49–59.

	Phase I (double-blind)					Phase II (single-blind)				
	Oxytocin		Placebo			Oxytocin$_{first}$		Placebo$_{first}$		
	N	Mean ± SD	N	Mean ± SD	*p*-value	N	Mean ± SD	N	Mean ± SD	*p*-value
**Week 1**										
**Informant report**										
Valence	37	3.10 ± 0.92	36	3.03 ± 1.17	0.780	36	3.74 ± 1.54	38	3.55 ± 1.23	0.565
Arousal	37	3.70 ± 1.13	36	3.64 ± 1.24	0.831	36	3.05 ± 1.03	38	3.30 ± 1.36	0.366
**Self report**										
Valence - noon	36	2.71 ± 1.15	35	2.68 ± 1.19	0.905	37	2.36 ± 1.11	39	2.67 ± 1.32	0.280
Arousal - noon	36	3.30 ± 1.20	35	3.20 ± 1.25	0.749	37	3.07 ± 1.51	39	3.27 ± 1.57	0.578
Valence - evening	36	2.69 ± 1.15	35	2.70 ± 1.13	0.957	37	2.25 ± 1.07	39	2.92 ± 1.32	**0.017**
Arousal - evening	36	3.47 ± 1.29	35	3.16 ± 1.07	0.279	37	3.10 ± 1.67	39	3.49 ± 1.48	0.283
**Week 2**										
**Informant report**										
Valence	37	3.23 ± 1.04	36	2.96 ± 1.32	0.323	36	3.55 ± 1.55	38	3.32 ± 1.11	0.461
Arousal	37	3.93 ± 1.45	36	3.55 ± 1.36	0.246	36	2.79 ± 0.96	38	2.99 ± 1.27	0.446
**Self report**										
Valence - noon	36	2.78 ± 1.25	35	2.31 ± 1.20	0.109	35	2.02 ± 0.94	38	2.41 ± 1.24	0.139
Arousal - noon	36	3.58 ± 1.66	35	3.00 ± 1.53	0.135	35	2.92 ± 1.71	38	2.89 ± 1.40	0.922
Valence - evening	36	3.01 ± 1.28	35	2.32 ± 1.15	**0.019**	35	2.32 ± 1.04	38	2.53 ± 1.21	0.441
Arousal - evening	36	3.70 ± 1.49	35	3.04 ± 1.38	0.057	35	3.20 ± 1.69	38	2.94 ± 1.37	0.477
**Week 3**										
**Informant report**										
Valence	37	3.19 ± 1.09	36	2.95 ± 1.08	0.345	37	3.83 ± 1.97	39	3.23 ± 1.41	0.124
Arousal	37	3.86 ± 1.38	36	3.32 ± 1.31	0.092	37	3.03 ± 1.24	39	2.80 ± 1.19	0.422
**Self report**										
Valence - noon	36	2.47 ± 1.15	34	2.25 ± 1.15	0.434	36	2.03 ± 1.04	38	2.29 ± 1.08	0.286
Arousal - noon	36	3.21 ± 1.47	34	3.01 ± 1.67	0.588	36	2.80 ± 1.68	38	2.62 ± 1.37	0.618
Valence - evening	36	2.73 ± 1.11	34	2.39 ± 1.27	0.230	36	2.20 ± 0.99	38	2.37 ± 1.15	0.504
Arousal - evening	36	3.26 ± 1.33	34	3.17 ± 1.73	0.801	36	2.96 ± 1.77	38	2.52 ± 1.24	0.226
**Week 4**										
**Informant report**										
Valence	35	3.11 ± 0.81	36	2.89 ± 1.26	0.383	37	3.63 ± 1.86	39	3.13 ± 1.33	0.179
Arousal	35	3.79 ± 1.35	36	3.23 ± 1.31	0.081	37	2.89 ± 1.28	39	2.79 ± 1.15	0.724
**Self report**										
Valence - noon	34	2.51 ± 1.09	34	2.23 ± 1.17	0.306	36	2.03 ± 0.98	38	2.08 ± 0.95	0.801
Arousal - noon	34	3.03 ± 1.37	34	2.91 ± 1.42	0.721	36	2.85 ± 1.74	38	2.57 ± 1.43	0.450
Valence - evening	34	2.72 ± 1.18	34	2.51 ± 1.25	0.467	36	2.18 ± 1.05	38	2.34 ± 1.10	0.533
Arousal - evening	34	3.35 ± 1.23	34	3.16 ± 1.65	0.607	36	3.07 ± 1.79	38	2.79 ± 1.45	0.462
**Across weeks**										
**Informant report**										
Valence	37	3.17 ± 0.70	36	2.96 ± 1.00	0.302	36	3.69 ± 1.60	38	3.26 ± 1.00	0.163
Arousal	37	3.83 ± 1.15	36	3.43 ± 1.06	0.131	36	2.94 ± 0.95	38	2.93 ± 1.05	0.969
**Self report**										
Valence - noon	36	2.62 ± 0.98	35	2.36 ± 1.10	0.282	37	2.18 ± 1.02	39	2.42 ± 1.09	0.333
Arousal - noon	36	2.79 ± 1.02	35	2.46 ± 1.02	0.175	37	2.24 ± 0.86	39	2.59 ± 1.06	0.120
Valence - evening	36	3.29 ± 1.23	35	3.01 ± 1.29	0.355	37	2.92 ± 1.54	39	2.88 ± 1.38	0.917
Arousal - evening	36	3.45 ± 1.14	35	3.11 ± 1.28	0.247	37	3.06 ± 1.62	39	2.99 ± 1.24	0.818

Valence scale: Higher scores denote feeling more unpleasant/unhappy (1= feeling pleasant/happy, 9= feeling unpleasant/unhappy). **Arousal scale:** Higher scores denote feeling more excited (1= calm, 9= excited).

Supplementary Table 5

Detailed description of questionnaires adopted as descriptive, primary or secondary outcomes.

Outcome measures	Construct	Type of outcome	Type of report	Number of items	Range of scores	Rating scale (points)	Meaning of higher scores	Reference
**Autism Diagnostic Observation Schedule (ADOS-2)**	Symptom severity	Descriptive	Observation	Per module	1-10	-	More severe symptoms of autism spectrum disorder	[1]
**Wechsler Intelligence Scale for Children (WISC-V-NL)**	Verbal Intelligence Quotient	Descriptive	Observation	Similarities Vocabulary	40 - 145	-	Higher verbal abilities	[2]
	Performance Intelligence Quotient			Block Design Visual Puzzles	40 - 145	-	Higher visual spatial abilities	
**Social Responsiveness Scale-Children (SRS-2)**	Symptom severity	Primary	Parent	65	0 - 192	0-3	Greater deficits in social responsiveness	[3], [4]
**Repetitive Behavior Scale-Revised (RBS-R)**	Repetitive behavior	Secondary	Parent	43	0 - 129	0-3	More severe repetitive behavior	[5], [6]
**Screen for Child Anxiety Related**	Anxiety	Secondary	Parent and self	69	0 - 207	0-2	Higher risk for anxiety disorders	[7]

**Emotional Disorders (SCARED-NL)**								
**Attachment Style Classification Questionnaire (ASCQ)**<br>**Anxious**<br>**Avoidant**<br>**Secure**	Attachment	Secondary	Self	15	Per subscale 5 - 25	1-5	More anxious, avoidant or secure attachment toward their peers	[8]
**Attachment questionnaire**<br>**Anxious**<br>**Avoidant**<br>**Secure**	Attachment	Secondary	Self	9	Per subscale 3 - 21	1-7	More anxiety, avoidance or trust toward their mother	[9]

References:

[1] Lord C, Rutter M, DiLavore PC, Risi S, Gotham K,, Bishop SL. Autism Diagnostic Observation Schedule: ADOS-2. Los Angeles: Western Psychological Services. 2012.

[2] Wechsler D. WISC-V-NL. Wechsler Intelligence Scale for Children, Fifth Edition, Dutch version. Amsterdam: Pearson Benelux B.V.; 2018.

[3] Constantino JN, Gruber CP. Social responsiveness scale (2nd. ed.). Manual. Los Angeles: Western Psychological Services; 2012.

[4] Roeyers H, Thys M, Druart C, de Schryver M, Schittekatte M. SRS-2: Screeningslijst voor autismespectrumstoornissen. Amsterdam: Hogrefe Uitgevers; 2015.

[5] Bodfish JW, Symons FJ, Parker DE, Lewis MH. Varieties of repetitive behavior in autism: comparisons to mental retardation. J Autism Dev Disord. 2000;30:237–43.

[6] Lam KSL, Aman MG. The Repetitive Behavior Scale-Revised: independent validation in individuals with autism spectrum disorders. J Autism Dev Disord. 2007;37:855–66.

[7] Muris P, Bodden D, Hale W, Birmaher B, Mayer B. SCARED-NL. Vragenlijst over angst en bang-zijn bij kinderen en adolescenten. Handleiding bij de gereviseerde Nederlandse versie van de Screen for Child Anxiety Related Emotional Disorders. Amsterdam: Boom Test Uitgevers; 2007.

[8] Finzi R, Cohen O, Sapir Y, Weizman A. Attachment styles in maltreated children: a comparative study. Child Psychiatry Hum Dev. 2000;31:113–28.

[9] Bosmans G, van de Walle M, Goossens L, Ceulemans E. (In)variability of attachment in middle childhood: Secure base script evidence in diary data. Behaviour Change. 2014;31:225–42.

Supplementary Table 6

Mean questionnaire scores for each treatment group and assessment session.

For each questionnaire, raw mean scores and standard deviations are listed separately for each treatment group (oxytocin, placebo) and each assessment session (baseline, post, follow-up) of each phase (phase I and II).

	Baseline				Phase I (double-blind)							
					Post				Follow-up			
	Oxytocin		Placebo		Oxytocin		Placebo		Oxytocin		Placebo	
**Outcome Measure**	**N**	**Mean ± SD**	**N**	**Mean ± SD**	**N**	**Mean ± SD**	**N**	**Mean ± SD**	**N**	**Mean ± SD**	**N**	**Mean ± SD**
**Primary Outcome**												
SRS informant based	38	89.26 ± 21.66	39	87.87 ± 20.03	38	85.18 ± 23.56	38	83.66 ± 21.88	38	82.50 ± 25.58	39	82.49 ± 23.88
**Secondary Outcomes - informant report**												
RBS	38	27.29 ± 15.24	39	26.64 ± 16.43	38	20.76 ± 13.27	38	19.50 ± 14.70	38	22.74 ± 14.19	39	22.23 ± 16.71
SCARED Parent	38	39.74 ± 21.74	39	45.15 ± 18.31	38	39.26 ± 21.51	38	40.16 ± 18.31	38	36.82 ± 21.08	39	39.77 ± 17.69
**Secondary Outcomes - self report**												
SCARED Child	38	38.29 ± 20.99	39	39.05 ± 20.21	38	33.55 ± 20.67	39	35.67 ± 21.18	38	32.32 ± 20.57	39	32.69 ± 17.85
ASCQ Secure	38	19.97 ± 3.50	39	19.23 ± 2.78	38	19.08 ± 3.24	39	18.31 ± 3.42	38	18.61 ± 3.38	39	18.41 ± 3.75
ASCQ Anxious	38	13.45 ± 5.19	39	12.85 ± 4.15	38	12.53 ± 5.00	39	11.85 ± 3.80	38	12.45 ± 5.06	39	10.46 ± 3.42
ASCQ Avoidant	38	13.79 ± 4.00	39	14.08 ± 3.86	38	13.50 ± 3.91	39	13.00 ± 4.01	38	12.95 ± 4.38	39	12.62 ± 3.81
Attachment Mother Anxiety	38	4.74 ± 2.89	39	4.82 ± 2.78	38	5.50 ± 2.68	39	4.49 ± 2.02	38	5.58 ± 2.90	39	4.90 ± 2.33
Attachment Mother Avoidance	38	9.05 ± 4.76	39	7.97 ± 4.03	38	8.84 ± 5.01	39	7.15 ± 2.80	38	8.45 ± 4.72	39	7.31 ± 3.29
Attachment Mother Secure	38	16.76 ± 4.24	39	17.87 ± 3.13	38	17.00 ± 3.57	39	17.69 ± 3.37	38	17.42 ± 3.55	39	18.00 ± 2.93

	Phase II (single-blind)							
	Post				Follow-up			
	Oxytocin$_{first}$		Placebo$_{first}$		Oxytocin$_{first}$		Placebo$_{first}$	
**Outcome Measure**	**N**	**Mean ± SD**	**N**	**Mean ± SD**	**N**	**Mean ± SD**	**N**	**Mean ± SD**
**Primary Outcome**								
SRS informant based	37	83.81 ± 25.14	38	77.37 ± 22.75	36	79.22 ± 24.28	36	78.14 ± 23.78
**Secondary Outcomes - informant report**								
RBS	37	20.57 ± 16.51	38	19.13 ± 16.66	36	19.58 ± 15.55	36	17.08 ± 12.76
SCARED Parent	37	37.43 ± 22.57	38	37.92 ± 18.96	36	33.81 ± 21.88	36	35.75 ± 17.17
**Secondary Outcomes - self report**								
SCARED Child	37	30.38 ± 20.47	39	31.82 ± 21.14	36	29.58 ± 20.36	37	29.19 ± 17.30
ASCQ Secure	37	18.32 ± 3.51	39	18.41 ± 3.85	36	18.19 ± 2.81	37	18.19 ± 3.41
ASCQ Anxious	37	12.24 ± 5.36	39	10.41 ± 3.75	36	11.83 ± 4.83	37	10.89 ± 4.21
ASCQ Avoidant	37	12.46 ± 3.58	39	12.77 ± 4.21	36	12.64 ± 3.94	37	13.24 ± 3.95
Attachment Mother Anxiety	37	5.70 ± 3.61	39	5.13 ± 3.29	36	5.72 ± 4.03	37	5.00 ± 2.99
Attachment Mother Avoidance	37	8.30 ± 5.06	39	6.85 ± 3.34	36	7.94 ± 5.23	37	7.16 ± 3.38
Attachment Mother Secure	37	16.97 ± 3.37	39	17.03 ± 4.20	36	17.14 ± 4.43	37	17.46 ± 3.12

SRS Social Responsiveness Scale, RBS-R Repetitive Behavior Scale-Revised, SCARED Screen for Child Anxiety Related Disorders, ASCQ Attachment Style Classification Questionnaire.

Supplementary Table 7

Effects of oxytocin nasal spray administration on primary and secondary outcome measures of the single-blind phase II.

Changes from assessment session T2 (last session of phase I) are listed separately for the post assessment session (immediately after the four-week oxytocin nasal spray administration period, T3) and the follow-up assessment (four weeks after cessation of the nasal spray administration period, T4).

	Within-group													Between-group		
	Oxytocin-first group							Placebo-first group								
**Outcome measure**	***n***	**mean**	**±**	**SD**	***t*-value**	***p*-value**	***n***	**mean**	**±**	**SD**	***t*-value**	***p*-value**	**Cohen's *d***	***t*-value**	***p*-value**
**T3 Post assessment**															
**Primary Outcome**															
SRS-2 total raw score	37	1.41	±	9.91	0.86	0.394	38	-5.24	±	9.87	-3.27	**0.002**	0.67	2.91	**0.005**
**Secondary Outcomes - parent report**															
RBS-R	37	-1.76	±	8.68	-1.23	0.226	38	-2.89	±	4.52	-3.94	**0.000**	0.17	0.71	0.477
SCARED Parent	37	0.27	±	7.31	0.22	0.823	38	-1.53	±	5.87	-1.60	0.117	0.27	1.18	0.244
**Secondary Outcomes - self report**															
SCARED Child	37	-2.76	±	10.53	-1.59	0.120	39	-0.87	±	11.78	-0.46	0.647	-0.17	-0.73	0.465
ASCQ Secure	37	-0.22	±	4.06	-0.32	0.748	39	0.00	±	2.14	0.00	1.000	-0.07	-0.29	0.771
ASCQ Anxious	37	-0.38	±	3.53	-0.65	0.519	39	-0.05	±	2.90	-0.11	0.913	-0.10	-0.44	0.660
ASCQ Avoidant	37	-0.70	±	3.40	-1.26	0.217	39	0.15	±	2.82	0.34	0.736	-0.27	-1.20	0.235
Attachment Mother Anxiety	37	0.08	±	3.06	0.16	0.873	39	0.23	±	2.64	0.55	0.588	-0.05	-0.23	0.820
Attachment Mother Avoidance	37	-0.30	±	3.13	-0.58	0.566	39	-0.46	±	2.27	-1.27	0.212	0.06	0.26	0.793
Attachment Mother Secure	37	-0.35	±	3.23	-0.66	0.513	39	-0.97	±	3.38	-1.80	0.080	0.19	0.82	0.415
**T4 Follow-up assessment**															
**Primary Outcome**															
SRS-2 total raw score	36	-2.94	±	11.09	-1.59	0.120	36	-3.53	±	9.11	-2.32	**0.026**	0.06	0.24	0.808
**Secondary Outcomes - parent report**															
RBS-R	36	-2.75	±	9.10	-1.81	0.078	36	-3.19	±	7.19	-2.67	**0.012**	0.05	0.23	0.819
SCARED Parent	36	-2.17	±	9.62	-1.35	0.185	36	-1.89	±	8.30	-1.36	0.181	-0.03	-0.13	0.896
**Secondary Outcomes - self report**															
SCARED Child	36	-4.03	±	13.36	-1.81	0.079	37	-4.51	±	11.22	-2.45	**0.019**	0.04	0.17	0.867
ASCQ Anxious	36	-0.39	±	3.41	-0.68	0.498	37	-0.41	±	2.51	-0.98	0.333	0.01	0.02	0.981
ASCQ Avoidant	36	-0.53	±	2.44	-1.30	0.203	37	0.32	±	3.21	0.62	0.542	-0.30	-1.27	0.207
ASCQ Secure	36	-0.36	±	3.30	-0.66	0.516	37	0.65	±	3.31	1.19	0.241	-0.31	-1.30	0.196

Attachment Mother Anxiety	36	0.11	±	3.87	0.17	0.864	37	0.00	±	2.95	0.00	1.000	0.03	0.14	0.890
Attachment Mother Avoidance	36	-0.53	±	3.14	-1.01	0.320	37	-0.19	±	2.75	-0.42	0.678	-0.11	-0.49	0.625
Attachment Mother Secure	36	-0.22	±	2.70	-0.49	0.624	37	-0.43	±	2.61	-1.01	0.320	0.08	0.34	0.736

SRS-2: Social Responsiveness Scale, RBS-R: Repetitive Behavior Scale-Revised, SCARED: Screen for Child Anxiety Related Disorders, ASCQ: Attachment Style Classification Questionnaire. For all outcomes, except ASCQ secure and Attachment mother secure, negative change from baseline scores indicate pre-to-post improvement. Values printed in bold indicate p-values < 0.05.
